# Electrocardiographic phenotype of exercise-induced arrhythmogenic cardiomyopathy: A retrospective observational study

**DOI:** 10.3389/fcvm.2022.1052174

**Published:** 2022-11-23

**Authors:** Hielko Miljoen, Francesco Spera, Katrien Van Kolen, Johan Saenen, Guido Claessen, Wim Huybrechts, Andrea Sarkozy, Hein Heidbuchel

**Affiliations:** ^1^Department of Cardiology, University Hospital, Antwerp, Belgium; ^2^Cardiovascular Research, Department GENCOR, University of Antwerp, Antwerp, Belgium; ^3^Klina Hospital, Brasschaat, Belgium; ^4^Department of Cardiology, University Hospital Leuven, Leuven, Belgium; ^5^Department of Cardiovascular Sciences, Cardiology, Leuven University, Leuven, Belgium

**Keywords:** electrocardiography, arrhythmogenic right ventricular cardiomyopathy (ARVC), ACM, athlete, phenotype, exercise

## Abstract

**Introduction:**

The right ventricle can be susceptible to pathologic alterations with exercise. This can cause changes to the ECG. Our aim was to identify the electrocardiographic phenotype of exercise induced (ExI) arrhythmogenic cardiomyopathy (ACM).

**Methods:**

A retrospective analysis of ECGs at rest, peak exercise and 1 min of recovery in four groups of individuals was performed: Arrhythmogenic Cardiomyopathy with genetic confirmation (Gen-ACM; *n* = 16), (genetically negative) ExI-ACM (*n* = 15), control endurance athletes (End; *n* = 16) and sedentary individuals (Sed; *n* = 16). The occurrence of ventricular arrhythmias (VA) and, at each stage, QRS duration, Terminal Activation Delay (TAD), the ratio of the sum of the QRS durations in the right precordials (V1-V3) over that in the left precordials (V4-V6; R/L duration ratio), the presence of complete RBBB and T-wave inversion (TWI) beyond lead V2 were evaluated.

**Results:**

At rest, complete RBBB was exclusively found in Gen-ACM (6%) and ExI-ACM (13%). No epsilon waves were identified. TWI beyond V2 was uniquely present in Gen-ACM (73%) and ExI-ACM (38%; *p* < 0.001). VA was present in Gen-ACM (88%); ExI-ACM (80%), End (25%) and Sed (19%; *p* < 0.001). The presence of R/L duration ratio of >1.2 and TAD ≥ 55 ms were not significantly different over the four groups (*p* = 0.584 and *p* = 0.218, respectively). At peak exercise the most striking finding was a significant decrease of the R/L duration ratio in individuals with ACM, which was the result of lateral precordial QRS prolongation.

**Conclusion:**

ExI-ACM shares important ECG-features with Gen-ACM, suggesting a similar underlying pathogenesis regardless of the presence or absence of desmosomal mutations.

## Introduction

Physical training for more than 3 h per week results in cardiac adaptations (1). Where traditionally the left ventricle was the focus of research, more recent work has drawn the attention to the vulnerability of the right ventricle (RV) for intense endurance loads. Indeed, regularly performing sports causes the phenotype of arrhythmogenic cardiomyopathy (ACM) to progress faster (2). Moreover, in a series of 46 high-level athletes (80% cyclists) with ventricular arrhythmias (VA), an important involvement of the RV was found (3). Although phenotypically similar to ACM, the prevalence of gene mutations causing ACM was lower than expected in this and a second, similar cohort of athletes (4, 5). This led to the hypothesis of a cardiomyopathy as the result of excessive wall stress during exercise, called “exercise-induced ACM” (ExI-ACM) (6).

One of the ECG traits of ACM with an identified desmosomal gene mutation (Gen-ACM) is activation delay over the RV (7). This can manifest as:

- Complete and incomplete right bundle branch block (cRBBB, iRBBB) (8).
- Epsilon waves (9–11).
- Terminal Activation Delay (TAD) ≥ 55 ms in the absence of cRBBB (12).
- Prolongation of the QRS duration in the right precordials compared to that in the left precordial leads [expressed as the ratio of the QRS complex duration in V1 + V2 + V3/V4 + V5 + V6, “R/L duration ratio” (>1.2)] (9).

Exercise can unmask some of these features, such as an prolongation of TAD, both in symptomatic ACM and in asymptomatic disease (13).

Our goal was to identify the ECG-features of ExInd-ACM at rest and during exercise, and compare those with Gen-ACM, non-ACM endurance athletes (End) and sedentary controls (Sed).

## Materials and methods

The study received approval from the ethical committee. We retrospectively gathered data on four groups of individuals: Gen-ACM, ExI-ACM, End and Sed. The latter two groups were selected from a consecutive series of patients older than 25 years presenting for a cardiologic screening (in a general preventive setting) and stratified for athletic performance. The following definitions were used:

- Endurance athletes: individuals participating in endurance sports (14) ≥4 h/week and having done so for ≥5 years (15).
- Sedentary individuals: otherwise healthy individuals exercising <2.5 h/week (15).
- Gen-ACM: definite ACM diagnosis according to the 2010 Task Force criteria in combination with the identification of a known ACM-gene (at least class 4 in the 5-class system) (12, 16). Those patients were identified from a single center patient database query using the following search terms: ACM, ARVC, and ARVD. Patients with clinical, genetic of imaging (Cardiac Magnetic Resonance) evidence of ACM were withheld for further evaluation.
- ExI-ACM: endurance athletes with an ACM phenotype (e.g., ventricular arrhythmias from the RV, CMR findings compatible with RV disease), in absence of a mutation in any of the known ACM-genes (17). Those patients were recruited from a previously described population of athletes with addition of the since then newly identified individuals fulfilling the same criteria (4).

An analysis of the exercise history was performed as described by Sawant et al. (18). In brief, exercise duration (in hours per week) was identified for each sport performed by an athlete. Each of these values was then multiplied by the appropriate MET value (Metabolic Equivalent) as read from the 2011 Compendium of Physical Activities to obtain MetHour per week for this sport (19). The results for all types of sports performed by the athlete were then summed and multiplied by 52 to obtain MetHours/year.

Genetic testing was performed in two BELAC accredited (ISO 15189:2012) expert cardiogenetic centers (Leuven and Antwerp University Hospitals) as described previously. In short, Genomic DNA was extracted from EDTA blood using standard procedures (Chemagic DNA bloodkit special, Perkin Elmer, Waltham, MA, USA). For ExI-ACM patients Sanger sequencing was performed with an ABI 3130XL Genetic Analyser (Applied Biosystems) in both directions using the BigDye Terminator cycle sequencing kit v3.1 (Applied Biosystems Inc, Foster City, CA, USA) and analyzed with the SeqMan II V.4.00 8 software (DNASTAR Inc., Madison, WI, USA) (4). For the Gen-ACM patients a Haloplex/Twist target enrichment followed by a next generation sequencing panel including all five desmosomal genes was applied (20). Variants were classified as benign or likely benign (class 1 and 2), variant of unknown significance (class 3), likely pathogenic (class 4) or pathogenic (class 5) according to ACMG guidelines (16).

In the ACM-groups the time from diagnosis to the analyzed exercise test was determined. ECG measurements were performed on digitized tracings by an experienced electrophysiologist (FS) blinded to the group assignments. With EP Calipers^®^ (EP Studios, Inc.^®^) an interval of 1,000 ms was measured as a reference. Three QRS complexes were evaluated per precordial lead. The average of these three measurements was used for further analysis. This process was completed for the ECG at rest, peak exercise and at 1 min recovery.

All ECGs were evaluated for the presence of cRBBB, iRBBB, an epsilon wave, T-Wave inversion (TWI) beyond V2 and VA. In individuals without cRBBB or Left Bundle Branch Block (i.e., QRS duration < 120 ms) during any exercise stage, additionally QRS durations and TAD (longest value in V1–3 from the nadir of the S wave (S_nadir_) to the end of all depolarization deflections) were measured (9).

Subsequently, screenshots of V1 (i.e., the lead with the largest variance in our series) were taken from the first 50 study patients, both at rest and at peak exercise. QRS duration was remeasured by three experienced electrophysiologists (FS, JS, and HM) as described above. From these data intra- and interobserver intraclass correlation coefficients (ICC) were calculated.

### Statistical analysis

For statistical analysis IBM SPSS statistics vs. 27.0.1.0 was used. A *p*-value of <0.05 was considered statistically significant.

Frequencies were compared using the Chi Square or Fisher's Exact Test as appropriate. Continuous parameters were tested for normal distribution using the Kolmogorov–Smirnov test. Normally distributed data were compared with one-way-ANOVA. When a significant difference was found, Tukey's honestly significant difference *post-hoc* test was used. In the case the data were not normally distributed the Kruskal-Wallis test was employed, with the Mann-Whitney U test as a *post-hoc* analysis for the analysis of differences between individual groups. Bonferroni correction was used to adjust for multiple testing.

For the analysis of evolution of R/L duration ratio and TAD values over the different exercise stages a repeated measures ANOVA was performed with exercise stage as within subject and diagnosis or ACM as between subject effect. *Post-hoc* analysis was done with Tukey's honestly significant difference test with Bonferroni correction for multiple testing. If relevant, receiver operating curve analysis (ROC) was performed and, based on the Youden index, the optimal cut-off value was determined. To control for the imbalance in gender and Amiodarone use (21) a *post-hoc* sensitivity analysis with mixed model ANOVA was performed.

To assess intraobserver variability intraclass correlation (ICC 1,1) was calculated based on a one-way random effects model. For the interobserver variability the intraclass correlation for absolute agreement based on a two-way random effects model was used (ICC 2,1) (22, 23).

## Results

**Figure 1:**
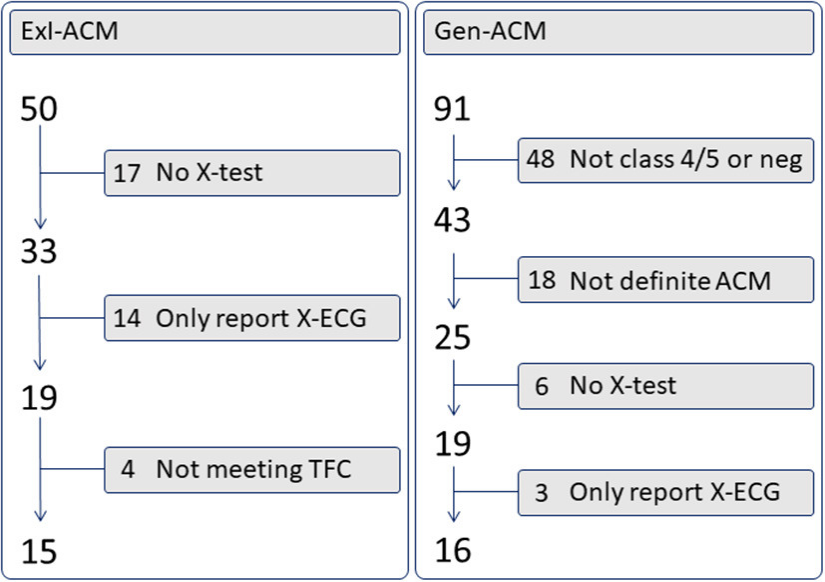
Recruitment flowchart ACM patients. No X-test, no exercise test was found in the medical record; Only report X-ECG, an exercise test had been performed, but the tracings could not be retrieved from the medical record; Not class 4/5 or neg, no class 4 or 5 genetic mutation was found (i.e., class 3 or lower, or none); Not meeting TFC, not meeting the 2010 Task Force Criteria as defined in the methods section.

Data on 63 individuals were collected: 16 Gen-ACM, 15 ExI-ACM, 16 End, and 16 Sed (Table 1). Figure 1 shows the recruitment flowchart for ExI-ACM and Gen-ACM patients. All individuals were in sinus rhythm and unpaced. In the ExI-ACM and End groups only males were present, while in the other groups also females were present.

**Table 1:** Baseline characteristics.

	**Gen-ACM**	**ExI-ACM**	**Endurance**	**Sedentary**	***p***
	**(*n* = 16)**	**(*n* = 15)**	**(*n* = 16)**	**(*n* = 16)**	
Gender (m/f)	10/6	15/0	16/0	14/2	0.003
Age at diagnosis (years)	40.6 (4.1)	43.0 (2.8)	44.9 (1.9)	46.2 (2.5)	0.554
Age at test (years)	42.2 (4.0)	50.1 (2.7)	44.9 (1.9)	46.2 (2.5)	0.287
Height (cm)	172.2 (2.2)^#^	178.8 (1.9)	181.8 (1.5)^§^	177.8 (1.9)	0.007
Weight (kg)	75.2 (3.2)	85.1 (2.3)	76.8 (1.6)	79.9 (3.6)	0.052
Endurance Sports	0	15	16	0	<0.001
MetHours/year	317 (122)	8,895 (1,541)	6,219 (495)	345 (222)	<0.001
**ARVC diagnosis**
Definite	16 (100)	7 (47)			<0.001
Borderline		5 (33)			
Possible		3 (20)			
Negative			16 (100)	16 (100)	
ICD [*n* (%)]	10 (63)	7 (47)	0 (0)	0 (0)	<0.001
**Medication [*****n*** **(%)]**
None	5 (31)	2 (13)	15 (94)	11 (69)	<0.001
Betablocker	6 (38)	5 (33)	1 (6)	5 (31)	
Sotalol	5 (31)	1 (7)	0 (0)	0 (0)	
Amiodarone	0 (0)	5 (33)	0 (0)	0 (0)	
Class Ic	0 (0)	2 (13)	0 (0)	0 (0)	

Abbreviations as in the text.

§ *p* < 0.05 vs. Gen-ACM;

# *p* < 0.05 vs. End.

As per the definitions, individuals in the ExI-ACM and End group performed significantly more MetHour/year than those in the sedentary groups. The difference between the values of the ExI-ACM and End groups did not reach statistical significance (8895 ± 1541 vs. 6219 ± 495, *p* = 0.118). The use of antiarrhythmic drugs was more prevalent in Gen-ACM and ExI-ACM than in the other groups. ICDs were present in 10 (63%) and 7 (47%) of patients in the Gen-ACM and ExI-ACM groups. The mutations that were found in the Gen-ACM group concerned PKP2 in 11 (69%), DSG2 in 3 (19%) and DSP in 2 (12%; Supplementary Table).

All patients in the Gen-ACM group fulfilled the Task Force Criteria of a “definite” diagnosis. In the ExI-ACM group this was the case for 7 (47%) of subjects. 5 (33%) classified as “borderline” and 3 (20%) as “possible.” All of the End and Sed group classified as “negative.”

The median time (IQR) between diagnosis and the analyzed exercise test was 0.1 (0.7) year in the Gen-ACM group and 9.7 (11.7) year in the ExI-ACm group (*p* = 0.026).

### Depolarization

Concerning **intra-ventricular conduction delay**, a cRBBB was present in three individuals at rest (1 Gen-ACM, 6% and 2 ExI-ACM, 16%; *p* = 0.182) and an iRBBB in 9 subjects (2 Gen-ACM, 13%; 3 End, 19%; 4 Sed, 25%; *p* = 0.243; Table 2). None of the participants developed a new cRBBB or LBBB during exercise or recovery.

**Table 2:** Frequencies of ECG findings.

	**Gen-ACM**	**ExI-ACM**	**End**	**Sed**	***p***
	**(*n* = 16)**	**(*n* = 15)**	**(*n* = 16)**	**(*n* = 16)**	
**Depolarization**
**cRBBB [*****n*** **(%)]**
Rest	1 (6)	2 (13)	0 (0)	0 (0)	0.182
**iRBBB [*****n*** **(%)]**
Rest	2 (13)	0 (0)	3 (19)	4 (25)	0.243
Peak	2 (13)	2 (13)	3 (19)	5 (31)	0.636
Rec	2 (13)	3 (20)	3 (19)	6 (38)	0.364
**Ventricular arrhythmias [*****n*** **(%)]**
Any	14 (88)	12 (80)	4 (25)	3 (19)	<0.001
Couplets	7 (44)	3 (20)	0 (0)	0 (0)	<0.001
Complex	3 (19)	3 (20)	0 (0)	0 (0)	0.058
	**Gen-ACM**	**ExI-ACM**	**End**	**Sed**	***p***
	**(*****n*** = 15)^*^	**(*****n*** = 13)^*^	**(*****n*** = **16)**	**(*****n*** = **16)**	
**Depolarization**
**R/L duration ratio** **>** **1.2 [*****n*** **(%)]**
Rest	0 (0)	1 (8)	1 (6)	0 (0)	0.584
Peak	0 (0)	0 (0)	1 (6)	1 (6)	1.000
Rec	0 (0)	0 (0)	3 (19)	0 (0)	0.054
**TAD** **≥55 ms [*****n*** **(%)]**
Rest	3 (20)	3 (23)	0 (0)	2 (13)	0.218
Peak	5 (33)	2 (15)	2 (13)	1 (6)	0.255
Rec	5 (33)	6 (46)	2 (13)	4 (25)	0.226
**Repolarization (** ***n*** **%)**
**TWI V3**
Rest	11 (73)	5 (38)	0 (0)	0 (0)	<0.001
Peak	3 (20)	5 (38)	0 (0)	0 (0)	0.002
Rec	7 (47)	5 (38)	1 (6)	0 (0)	<0.001
**TWI V4**
Rest	7 (47)	5 (38)	0 (0)	0 (0)	<0.001
Peak	2 (13)	6 (46)	0 (0)	0 (0)	0.003
Rec	5 (33)	5 (38)	0 (0)	0 (0)	0.001

Results are reported as number of individuals (% of total). Rec, recovery; other abbreviations as in the text.

* Patients with cRBBB were excluded from the analysis.

A new iRBBB developed in three individuals: 2 ExI-ACM (13%) and 1 Sed (6%).

In none of the patients an **epsilon wave** was found during any of the exercise stages.

An **R/L duration ratio** >1.2 was found in three individuals at rest: 1 ExI-ACM (8%), one End (6%) and 1 Sed (6%; *p* = 0.584; Table 2). At peak exercise, two individuals had an R/L duration ratio >1.2: 1 End (6%) and 1 Sed (6%; *p* = 1.000).

**Figure 2:**
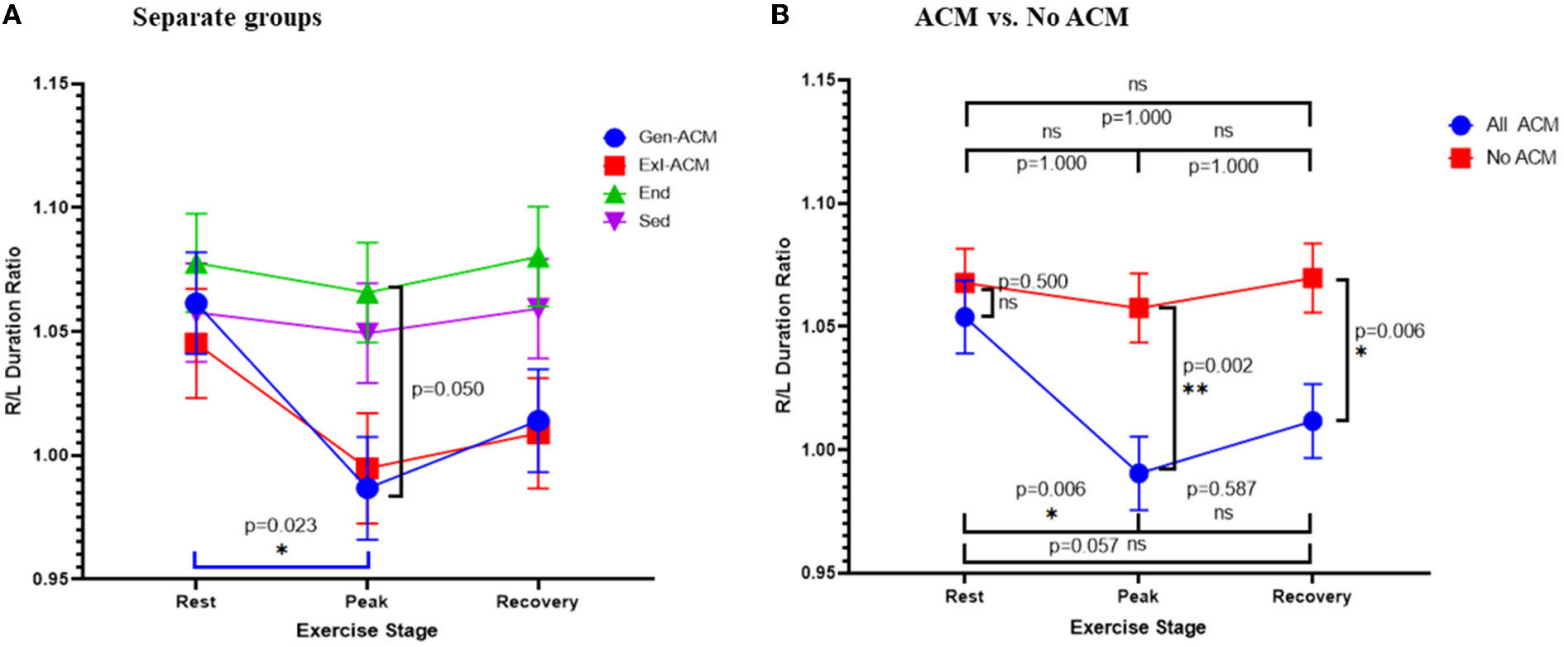
R/L duration ratio: ratio of the QRS duration V1 + V2 + V3 over QRS duration V4 + V5 + V6. R/L duration ratio over exercise stage classified according to diagnosis **(A)** and the presence or absence of ACM **(B)**. Error bars represent SE. *p < 0.05, **p < 0.01.

Analysis of the differences between the diagnostic groups for the R/L duration ratio as a continuous variable demonstrated a statistically significant difference of the values at rest and at peak in the Gen-ACM group (1.061 ± 0.020 vs. 0.987 ± 0.021 *p* = 0.023; Table 3A; Figure 2A). When the same was done for the pooled data of those with and those without evidence of ACM this was confirmed: those with ACM had a lower value at peak exercise than that at rest (0.991 ± 0.015 vs. 1.054 ± 0.014; *p* = 0.006). Furthermore, both at peak exercise and during recovery those with ACM had lower R/L duration ratios than those without (peak: 0.991 ± 0.015 vs. 1.057 ± 0.014; *p* = 0.002 and recovery: 1.012 ± 0.015 vs. 1.070 ± 0.014; *p* = 0.006; Table 3B; Figure 2B). The sensitivity analysis confirmed the importance of the presence of ACM (p_ACM_ = 0.009) and exercise stage (p_stage_ = 0.012), while Amiodarone use (*p* = 0.103) and gender (*p* = 0.543) did not influence this result.

**Table 3:** Depolarization parameters.

	**GenACM**	**ExI-ACM**	**End**	**Sed**	***P*_diagn_**	***P*_stage_**	***P*_diagnxstage_**
	**(*n* = 15)**	**(*n* = 13)**	**(*n* = 16)**	**(*n* = 16)**			
**(A) Stratified according to diagnosis**							
**R/L duration ratio**
Rest	1.061 (0.020)	1.045 (0.022)	1.078 (0.020)	1.058 (0.020)	0.157	0.015	0.451
Peak	0.987 (0.021)^$^	0.995 (0.022)	1.066 (0.020)	1.049 (0.020)			
Rec	1.014 (0.021)	1.009 (0.022)	1.080 (0.020)	1.059 (0.020)			
**TAD (ms)**
Rest	50.4 (2.2)	50.6 (2.4)	49.3 (2.2)	49.8 (2.1)	0.676	0.172	0.960
Peak	49.6 (1.9)	50.2 (2.0)	47.0 (1.8)	48.6 (1.8)			
Rec	52.1 (2.4)	52.8 (2.6)	48.6 (2.3)	49.6 (2.3)			
	**ACM**+		**ACM-**		*P*_ACM_	*P*_stage_	*P*_ACMxstage_
	**(*****n*** = **31)**		**(*****n*** = **35)**				
**(B) Stratified according to the presence or absence of ACM**							
**R/L duration ratio**
Rest	1.054 (0.014)		1.068 (0.014)		0.003	0.012	0.068
Peak	0.991 (0.015)^$^		1.057 (0.014)^*^				
Rec	1.012 (0.015)		1.070 (0.014)^*^				
**Prec (ms)**
Rest	322.9 (5.6)		312.1 (5.3)		0.167	0.001	0.748
Peak	308.1 (5.7)^$^		301.6 (5.4)				
Rec	317.5 (5.8)		306.3 (5.4)				
**Lat (ms)**
Rest	307.4 (5.4)		293.0 (5.1)		<0.001	0.841	0.059
Peak	311.8 (5.4)		286.0 (5.1)^*^				
Rec	314.3 (5.0)		286.8 (4.7)^*^				

Data are reported as mean (SE). p_*diagn*_, significance of the analysis between the diagnostic groups; p_*stage*_, significance of the analysis between the stages; p_*diagnxstage*_, significance of the interaction between diagnosis and stage; p_*ACM*_, significance of the analysis between ACM and no ACM; p_*ACMxstage*_, significance of the interaction between ACM and stage. Other abbreviations as in the text. ^*$*^*p* < 0.05 vs. rest; *p* < 0.05 vs. peak;

* *p* < 0.05 ACM+ vs. ACM−.

ROC analysis with calculation of the Youden index showed an optimal cut-off of 1.006 for the detection of any ACM through the R/L duration ratio at peak exercise, with a sensitivity of 0.64 (0.46–0.807) and a specificity of 0.78 (0.62–0.90). The C-statistic was 0.717 (SE: 0.066, *p* = 0.004).

**Figure 3:**
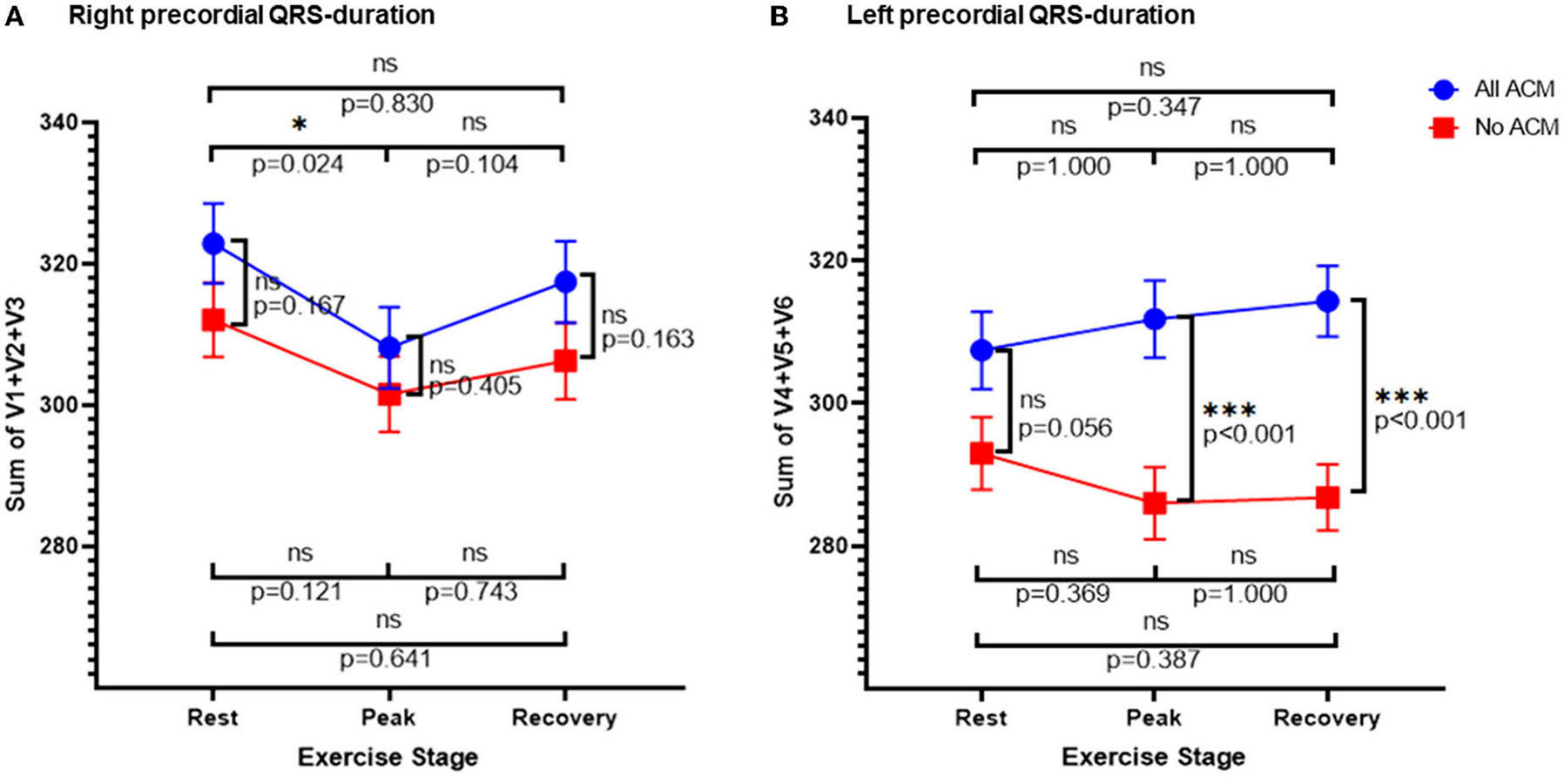
Right and left precordial QRS duration. Sum of right **(A)** and left **(B)** precordial QRS durations over the exercise stages stratified according to the presence or absence of ACM. Error bars represent SE. *p < 0.05, ***p < 0.001.

To further evaluate the explanation for the (paradoxically) lower R/L duration ratio at peak exercise in ACM, the sums of the QRS durations in the right and left precordial leads were analyzed separately (Table 3B; Figure 3). The right precordial leads showed a similar evolution over the exercise stages in subjects with and without ACM. Conversely, for the left precordials ACM patients showed an increase of the QRS-duration sum, while in those without ACM a shortening occurred, giving rise to a significant difference at peak exercise and recovery between those groups.

Terminal Activation Delay **(TAD)** ≥55 ms at rest was found in 8 subjects, showing no significant difference over the diagnostic groups: 3 Gen-ACM (20%), 3 ExI-ACM (23%) and 2 Sed (13%; *p* = 0.218). At peak exercise TAD ≥ 55 ms was found in 10 subjects: 5 Gen-ACM (33%), 2 ExI-ACM (15%), 2 End (13%) and 1 Sed (6%; *p* = 0.255). The findings were similar when pooled for presence or absence of ACM (*p* = 0.130 and *p* = 0.165, respectively). The same was true for the results of the analysis of TAD as a continuous variable for the diagnostic groups (Table 3A) and for the presence of absence of ACM.

The sensitivity and specificity of a TAD ≥ 55 ms at rest for the presence of RV disease were 0.21 (0.09–0.39) and 0.94 (0.82–0.99), respectively.

### Repolarization

**TWI** up till at least V3 was uniquely identified in ACM patients (11 Gen-ACM, 73%; 5 ExI-ACM 38%; *p* < 0.001). The same was true for TWI up till at least V4 (7 Gen-ACM, 47%; 5 ExI-ACM, 38%; *p* < 0.001). TWI till V3 had a sensitivity of 0.57 (0.39–0.74) and a specificity of 1.00 for the presence of RV disease in this study. For V4, this was 0.43 (0.29–0.61) and 1.00, respectively. The presence of TWI decreased at peak exercise and recovery (Table 2).

### Ventricular arrhythmias

Any type of **VA** (from single VBP to NSVT) during the exercise test occurred in 37 individuals: 14 Gen-ACM (88%), 12 ExI-ACM (80%), 4 End (25%) and 3 Sed (19%; *p* < 0.001). The presence of at least single VPB had a sensitivity of 0.84 (0.69–0.94) and a specificity of 0.79 (0.63–0.90) for the presence of any ACM. Couplets only occurred in those with ACM (7 Gen-ACM, 44%; 3 ExI-ACM, 20%; *p* < 0.001). More complex VA (triplets, NSVT) also exclusively occurred in the RV disease groups (3 Gen-ACM, 19%; 3 ExI-ACM, 20%; *p* = 0.058).

Of those individuals with VA, LBBB morphology was found in 13 Gen-ACM (93%), 13 ExI-ACM (92%), 1 End (25%) and 2 Sed (67%). The remaining individuals with VA showed only RBBB morphology. Of those with LBBB-VA, 5 Gen-ACM (36%), 4 Exi-ACM (17%), 1 End (25%) and 1 Sed (33%) also showed RBBB-VA. Superior-axis LBBB-VA were present in 10 Gen-ACM (71%), 4 ExI-ACM (33%), and 1 Sed (33%). Inferior axis LBBB-VA were present in 5 Gen-ACM (36%), 5 ExI-ACM (42%), 1 End (25%) and 1 Sed (33%). Two Gen-ACM patients had both superior and inferior axis LBBB VA. Bidirectional couplets were uniquely present in ACM patients: 5 Gen-ACM (36%) and 2 ExI-ACM (17%).

### Reproducibility

The ICC for intraobserver variance of the measurement of QRS duration in V1 at rest was 0.779 (0.641–0.868) and for interobserver variance 0.711 (0.548–0.823). For the same measurements at peak exercise these values were 0.750 (0.599–0.850) and 0.643 (0.477–0.780), respectively.

## Discussion

In our series the electrocardiographic phenotype of ExI-ACM matches that of Gen-ACM in many aspects, in spite of the absence of (currently known?) gene mutations:

- Complete RBBB was exclusively present in Gen-ACM (6%) and ExI-ACM (13%). The prevalence of a cRBBB in our group with Gen-ACM was similar to that reported by Peters (6%) and Cox (8%) (9, 11).
- In Gen-ACM the prevalence of TWI in V3 was 73% and in V4 47% and in ExI-ACM 38% for both. Our findings in Gen-ACM are comparable to the findings of Peters, Nasir and Cox who found, respectively, 54, 59, and 67% for V3 and 23 and 18% (Cox not reported) for V4 (9–11).
- The same was observed for VA: in Gen-ACM and ExI-ACM VA were much more prevalent and certainly more complex than in End and Sed. This also is in concordance with many previous observations (7). Indeed, the presence of LBBB morphology ventricular arrhythmias is, depending on the axis, considered a minor (inferior or indeterminate axis) or major (superior axis) criterion for the diagnosis of ACM (12).
- Epsilon waves were not found in our series. This is in line with data from previous work, which show a large interobserver variability for the diagnosis of epsilon waves (24).
- An R/L duration ratio >1.2 was rare in our series. Previous series reported prevalences of 98% (11), 77% (10), and 35% (9). It is clear that there is a large variation in the reported prevalences: from our 0% in Gen-ACM to Cox's near-100%. A first potential explanation for these differences might be a variation in disease severity. This, however, seems unlikely: as described above, the prevalence of cRBBB, TWI and VA were similar over the different series. A more plausible explanation for the variation in the reported prevalences is the reproducibility of the QRS duration measurements: for measurements at rest the ICC for intra- and interobserver variability was 0.779 (0.641–0.868) and 0.711 (0.548–0.823), respectively. At peak exercise this was 0.750 (0.599–0.850) and 0.643 (0.477–0.780). Although these ICC values suggest a “moderate” reliability, correct interpretation requires taking the confidence intervals into account (23). Based on these, at rest, both intra- and interobserver reliability are moderately reliable. At peak exercise, however, while the intraobserver reliability remains moderate, the interobserver reliability drops to poor. This is in line with the findings of Saguner et al. (25) who reported that interobserver correlations were higher for repolarization than for depolarization abnormalities.
- A similar reasoning holds true for a TAD ≥ 55 ms: we found this in the 20% of the Gen-ACM group, while Nasir, Cox and Perrin found prevalences of 95, 71, and 45%, respectively (9, 10, 13). Perrin et al. (13) found TAD-prolongation with exercise in 67% and 31% of symptomatic and asymptomatic ACM, respectively, where we observed this in only 2 Gen-ACM patients (13%). As demonstrated above, measurements during exercise pose even more difficulties than those at rest.
- The most striking finding from our data was the paradoxically low R/L duration ratio at peak exercise in patients with ACM. This resulted mainly from QRS-widening in the lateral leads, while there was no QRS-widening but rather -shortening in the right precordial leads. This may point to the well-known left ventricular involvement of ACM (5, 26, 27). Data from the Swiss ARVC registry demonstrated that patients with later onset LV involvement were more frequently competitive athletes compared to those with RV only or initial biventricular involvement (28). Another series of genetically negative athletes with RV arrhythmias also demonstrated that those with more severe arrhythmias showed more LV involvement (17). These findings certainly require further evaluation in future research.

The similar presentation of the two conditions (Gen-ACM and ExI-ACM) is in concordance with the strain-strainability continuum: Heidbuchel et al. (29) development of ACM depends on the strain (amount of exercise, other factors) vs. the tolerance of the ventricle(s) to sustain this strain (genetics). The ACM-threshold may be influenced by a monogenic trait, such as in Gen-ACM, polygenic susceptibility, or additional yet unknown factors like inflammation or (auto)immune response (30).

## Limitations

This is a retrospective study with all its inherent limitations. One of the most important drawbacks of the present study is the low number of patients in each group (partly due to the difficulty to retrospectively gather all relevant data on the diseased subjects). Although efforts were made to match the groups as best as possible, there were some significant differences between the four groups, notably regarding gender and use of AAD. This issue was addressed by performing a sensitivity analysis correcting for gender and amiodarone use producing similar results as the main analysis. Class IC drugs were rarely used in our series.

Secondly, in the ExI-ACM group the fulfillment of the 2010 Task Force Criteria was “definite” in 7(47%) of subjects. 5 (33%) classified as “borderline” and 3 (20%) as “possible.” This poses a risk of overdiagnosis of ACM in this group. Indeed a “gray zone” between physiological adaptation and pathological remodeling (whether electrical or morphological) has been described in athletes (31). Potentially some “healthily adapted” athletes have thus been included in our series. The major effect one would expect from this would be a dilution of pathological findings. In spite hereof the ECG similarities between Gen-ACM and ExI-ACM did surface from our data. One important conclusion, however, should be drawn from this: finetuning of both the criteria for Gen-ACM and ExI-ACM from ongoing research is still desirable. Potentially the advent of artificial intelligence and deep learning could prove helpful in this area.

As a result of the retrospective nature of our study, we observed a considerable difference in the time from the diagnosis of ACM to the analyzed exercise test between the Gen-ACM and the ExI-ACM groups (median 0.1 year vs. 9.7 years). All ExI-ACM patients were advised to refrain from participation in competitive sports or leisure-time activities of moderate to high intensity from the moment of their diagnosis (32). Where in reality this advice is at best followed moderately (44% of athletes did not adhere to this advice in a recent series) (33), this might hold implications for our results. Exercise exposure is known to be associated with a higher prevalence of an ACM-phenotype and VA in genotype positive family members of Gen-ACM probands (34) and clinical detraining was associated with a decrease in PVC burden in athletes with an ACM phenotype (whether Gen or ExI) (35). Continuation of exercise during the period between diagnosis and exercise testing therefore may worsen the clinical presentation, where refraining from exercise might result in a milder phenotype (36). Contrary to these findings, Costa et al. (33) recently demonstrated that where non-adherence to the sports advice did associate with a decrease in physical capacity over time, there was no significant effect on VA or functional parameters. We cannot exclude an influence of the time delay (progress in natural history, effects of sports prohibition) on our findings.

The reproducibility of the ECG measurements was at best moderate. One might postulate that the method we used is flawed. In other reported series, a variety of measuring methods were used that were certainly not more reproducible: Peters et al. (11) did not report their method, but since their work dates from 2003 they probably used a manual, non-digitized method. Cox et al. (9) also measured non-digitized measurements. Only Nasir et al. (10) used a 2-fold amplification and digital calipers, as we did. This shows that there is currently no “gold standard” for such measurements. The quality of a measuring tool can be judged from the intra- and interobserver variability. Here, again, a wide range of methods has been reported. Nasir et al. (10) reported a “measure of repeatability” on 20 ECGs, expressed as percentages, with intraobserver and interobserver differences of 1–6 and 1–7%. If we calculate these values we find up to 33% for intra- and up to 57% for interobserver differences. Of note, also Sarubbi et al. (37) reported values of 33 and 54.5%. Importantly, the expression of intra- and interobserver differences as percentages is a rather crude method. The more desirable method is the use of ICC (23). In this context, already in 1996 an investigation on reproducibility of manual measurement of the QRS interval reported similar values as we do, with an ICC for interobserver variability of 0.60 (0.53–0.66) (38). The main conclusion that can be drawn from these observations is that both for the measurement method and the reporting of intra- and interobserver variability, uniformity should be seeked, and that duration-dependent criteria have limited reliability.

## Conclusion

Our data demonstrate that ExI-ACM shares several ECG-features with Gen-ACM. This might add to the hypothesis of the strain-strainability continuum, where the balance of load and the tolerance for this load results in a similar phenotype even in the absence of manifest underlying desmosomal mutations (29).

The finding of a paradoxical prolongation of the QRS-duration in the left precordials might point to a higher than expected left ventricular involvement. These findings might heighten clinical awareness for this condition.

## Brief summary

Analysis of ECGs at rest, at peak exercise and after 1 min of recovery in four groups (sedentary individuals with genetically proven ACM, endurance athlete with genetically negative ACM phenotype, healthy endurance athlete and healthy sedentaries) demonstrated that a complete RBBB, T-wave inversion beyond V3 and the presence of Ventricular Arrhythmias were predominantly present in the two ACM groups, suggesting a similar mechanism of disease. Conversely, terminal activation delay and parietal block were not discriminative.

## Data availability statement

The raw data supporting the conclusions of this article will be made available by the authors, without undue reservation.

## Ethics statement

The studies involving human participants were reviewed and approved by the Ethics Committee of the Antwerp University Hospital, Drie Eikenstraat 655, 2650 Edegem, Belgium. Written informed consent for participation was not required for this retrospective study.

## Author contributions

HM, KV, and HH: conceptualization. HM, KV, JS, and HH: methodology. HM and FS: validation. HM: formal analysis, visualization, and writing - original draft. HM, FS, KV, and JS: investigation. HM, FS, KV, JS, and HH: resources. HM and FS: data curation. HM, FS, KV, JS, GC, WH, AS, and HH: writing-review and editing. GC and HH: supervision. All authors contributed to the article and approved the submitted version.

## Conflict of interest

HH received personal fees from Biotronik, Pfizer-BMS, and Daiichi-Sankyo. He received unconditional research grants through the University of Antwerp and/or the University of Hasselt from Bayer, Boehringer-Ingelheim, Bracco Imaging Europe, Abbott, Medtronic, Biotronik, Daicchi-Sankyo, Pfizer-BMS, and Boston-Scientific, all outside the scope of this work. HM received personal fees from Pfizer-BMS and Abbott, both outside the scope of this work. The remaining authors declare that the research was conducted in the absence of any commercial or financial relationships that could be construed as a potential conflict of interest.

## Publisher's note

All claims expressed in this article are solely those of the authors and do not necessarily represent those of their affiliated organizations, or those of the publisher, the editors and the reviewers. Any product that may be evaluated in this article, or claim that may be made by its manufacturer, is not guaranteed or endorsed by the publisher.
