# Supplementary material for: Electrocardiographic phenotype of exercise-induced arrhythmogenic cardiomyopathy: A retrospective observational study
Source: Front Cardiovasc Med. 2022 Nov 23;9:1052174. doi: 10.3389/fcvm.2022.1052174 (PMC9726729; doi:10.3389/fcvm.2022.1052174)
Supplement: Supplementary file 1 [file Table_1.pdf]

Supplementary Table
Genetic Variants in Gen-ACM Patients

	Genetic Variant	N
**PKP2**	c.1211dup, p.(Val406Serfs*4)	4
	c.2509delA, p.(Ser837Valfs*94)	3
	p.Y221X, (c.663C>A)	2
	4bp deletion (c.148_151delACAG, p.(Thr50Serfs))	1
	c.2489+1G>A intron 12	1
**DSG2**	c.145C>T, p.(Arg49Cys)	2
	c.689_690delAG, p.(Glu230Glyfs*37)	1
**DSP**	c.5008C>T, p.(Gln1670*)	1
	c.1670_1671delTA, p.(Ile557Argfs*27)	1

PKP2 plakophilin-2, DSG2 desmoglein-2, DSP desmoplakin. N: number of patients with mutation.
